# Duration and intensity of fluconazole for prophylaxis in preterm neonates: a meta-analysis of randomized controlled trials

**DOI:** 10.1186/s12879-016-1645-5

**Published:** 2016-06-27

**Authors:** Datian Che, Hua Zhou, Te Li, Bin Wu

**Affiliations:** Department of Respiratory Medicine, Shanghai Children’s Hospital, Shanghai Jiao Tong University, Shanghai, China; Department of Emergency Care, Wuxi Children’s Hospital, Affiliated with Nanjing Medical University, Wuxi, China; Department of Pharmacy, Yuxi People’s Hospital, Affiliated with the Kunming Medical College, Nieer Road 21, Yuxi, China; Medical Decision and Economic Group, Department of Pharmacy, Ren Ji Hospital, South Campus, School of Medicine, Shanghai Jiaotong University, Shanghai, China

**Keywords:** Fluconazole, Prophylaxis, Preterm neonates, Meta-analysis

## Abstract

**Background:**

The currently available evidence shows fluconazole is an effective prophylaxis treatment against invasive fungal infections in preterm neonates in neonatal intensive care units (NICUs). However, the duration and dosing of this prophylaxis treatment remain controversial. Thus, a meta-analysis and systematic review are necessary.

**Methods:**

PubMed and EMBASE were systematically searched with no restrictions. All relevant citations that compared prophylactic fluconazole and no prophylaxis were considered for inclusion. Pooled effect estimates were obtained through fixed- and random-effects meta-analyses, and a meta-regression was used to explore the sources of heterogeneity in the data.

**Results:**

Five independent randomized controlled clinical trials (RCTs) involving 1006 preterm neonates were identified. Compared with no prophylaxis, the overall combined relative risks (RRs) of invasive fungal infection with the 28- and 42-day durations of prophylactic fluconazole were 0.80 (95 % CI 0.48–1.35, *p* = 0.4048) and 0.30 (95 % CI 0.15–0.58, *p* = 0.0004), respectively. The fluconazole dose had no significant impact on the RR of invasive fungal infections. The RR of mortality presented no significant differences between prophylactic fluconazole and no prophylaxis (RR 0.82, 95 % CI 0.60 to 1.12, *p* = 0.2093).

**Conclusions:**

Prophylaxis with fluconazole for 42 days was found to be superior to no prophylaxis as a strategy for preventing invasive fungal infection in preterm infants in NICUs except in terms of mortality. The dosing regimen of prophylactic fluconazole may have no impact on the outcome; however, due to the limitations of the available data, further research is needed.

## Background

The prevalence of invasive fungal infections accounts for 10 % of all cases of late-onset infection (occurring at least 72 h after birth) in very-low-birth-weight (VLBW) preterm neonates (<1500 g) [1]. Due to their incomplete immunity and exposure to required invasive procedures and medications (e.g., broad-spectrum antibiotics, parenteral nutrition, H2 blockers and corticosteroids), preterm neonates are at high risk of invasive fungal infections, particularly infections with Candida species [2, 3]. The consequences of invasive fungal infections are severe. The reported mortality rates are 21–32 % in VLBW infants and 40–50 % in extremely-low-birth-weight (ELBW, i.e., <1000 g) infants [4, 5]. Significantly higher incidences of short-term morbidity (retinopathy of prematurity, chronic lung disease and periventricular leukomalacia) and long-term complication (neurodevelopmental impairment) have been reported in preterm infants who develop invasive fungal infections compared with infants without invasive candidiasis [6, 7]. The challenge is to establish adequate prevention strategies for such vulnerable populations and has resulted in the establishment of prevention strategies that aim to reduce the incidence of invasive fungal infection in preterm neonates.

A broad chemo-prophylactic strategy with fluconazole has shown efficacy, safety and long-term neurodevelopmental outcomes in neonatal intensive care units (NICUs) with a high burden (≥15 %) of candidiasis, as demonstrated in both randomized controlled trial (RCT) and non-RCT studies [8–14]. Austin and McGuire’s recent systematic review, which included seven trials involving 880 infants, concluded that prophylaxis with fluconazole or nystatin may be beneficial over no prophylaxis in terms of the incidence of invasive fungal infections but that it is unlikely to produce a better outcome in terms of mortality [15]. Clinical practice guidelines suggest the administration of prophylactic fluconazole for neonates with a birth weight of less than 1000 g in NICUs [16]. A recent study found that 42 days of prophylactic fluconazole resulted in a 6 % (95 % CI, 1 % to 11 %, *P* = 0.02) decrease in the incidence of invasive candidiasis in premature infants with a birth weight of less than 750 g. However, this treatment did not decrease mortality or neurodevelopmental impairment [17].

Although these studies indicate that prophylactic fluconazole is both safe and effective for preventing invasive fungal infections, the obvious differences in their comparative efficacy profiles remain. These differences include the duration of prophylaxis and the dosing regimen of fluconazole, which should be assessed. The current meta-analysis aimed to quantitatively pool the results of head-to-head RCTs to examine whether an increased duration of prophylaxis or an increased dosing of fluconazole would improve the outcomes of prophylaxis with fluconazole compared with no prophylaxis in preterm neonates in the NICU.

## Methods

### Search strategy

This analysis was performed according to the preferred reporting items for systematic reviews and meta-analyses (PRISMA) guidelines and the methods described in the Cochrane Handbook [18]. Two investigators (DC and HZ) independently searched all eligible studies in the PubMed and EMBASE databases until November 2014, and no specific restrictions on language or publication year were applied. The electronic search strategy included the terms (“preterm” OR “prematur*” OR “very low birth weight”) and (“azole,” OR “imidazole,” OR “fluconazole”) combined with “trial”. The titles and abstracts were scanned to exclude any clearly irrelevant studies. Furthermore, to identify any additional published reports, a manual search of all references in the original reports was performed. In addition, the reference lists of eligible studies in Google Scholar were reviewed to ensure that all appropriate studies were included. The results were compared, and any questions or discrepancies were resolved through iteration and consensus.

This study constitutes an analysis of published data and thus did not require ethics committee approval.

### Inclusion criteria

The following preselected criteria justified inclusion in this meta-analysis: (1) comparative RCT study; (2) neonates weighing less than 1500 g at birth; (3) head-to-head comparison of results between fluconazole and placebo; and (4) full-text manuscript available in a peer-reviewed journal.

### Data extraction and quality assessment

Two independent researchers (DC and HZ) evaluated all retrieved titles and abstracts for eligibility, and any disagreements were resolved by consensus. Duplicate studies (i.e., those found in more than one database) were removed. To select between studies that used the same cohort of participants, the study with the most comprehensive data was used in the meta-analysis. The following data were extracted: first author’s last name, year of publication, study design, number of patients, intervention, injection times, follow-up days (Table 1), efficacy, and adverse event information. The included studies were critically evaluated using the Jadad composite scale, which scores studies based on their descriptions of randomization (two points), blinding (two points), and attrition information (one point) [19]. If the above data were not available in the published study, the authors were contacted and asked to supply the information.

**Table 1:** Characteristics of randomized controlled trials included in the meta-analysis

Study	Preterm neonate	Number of neonates used in the analysis		Duration of prophylaxis (days)	Regimen	Average dosage of fluconazole (mg) /kg/day	Total dosage of fluconazole (mg)/kg	Research site	JADAD score
		Fluconazole	Control						
Kaufman, 2001 [8]	ELBW	50	50	42	3 mg/kg every 3 days for weeks 1–2, every 2 days for weeks 3–4, and daily for weeks 5–6	1.83	77	Single	5
Kicklighter, 2001 [9]	VLBW	53	50	28	6 mg/kg every 3 days for week 1 and daily for weeks 2–4	5.00	140	Single	5
Manzoni, 2007 [10]	VLBW	216	106	28 or 42	3 or 6 mg/kg every 3 days for weeks 1–2, every 2 days for weeks 3–4 in VLBW, and every 2 days for weeks 3–6 in ELBW.	NA	NA	Multi	5
Parikh, 2007 [11]	VLBW	60	60	28	3 mg/kg every 3 days for week 1 and every day for weeks 2–4.	2.50	70	Single	5
Benjamin, 2014 [17]	ELBW	188	173	42	3 mg/kg every 3 days for weeks 1–2, every 2 days for weeks 3–4, and daily for weeks 5–6.	1.71	72	Multi	5

*ELBW* extremely-low-birth-weight, *VLBW* very-low-birth-weight, *NA* not applicable

Invasive fungal infections and mortality rate were the primary outcomes studied in the analysis.

### Statistical analysis

The pooled relative risks (RRs) with 95 % CIs between prophylaxis with fluconazole and no prophylaxis were used to estimate the effect sizes using the ‘metafor’ and ‘meta’ packages, respectively, in R version 3.1.2 for Windows (The R Foundation for Statistical Computing, Vienna, Austria). The statistical heterogeneity among the studies was assessed using Cochran’s Q test and the I^2^ statistic [20]. A value of I^2^ > 50 % was considered to indicate substantial heterogeneity, and a *P* value <0.05 was considered to indicate significant heterogeneity [21]. Fixed-effects models were employed in the case of low heterogeneity (I^2^ < 30 %); otherwise, random-effects models were used. To assess whether publication bias may have impacted the statistical results, a funnel plot was created, and Egger’s and Begg’s tests were conducted [22, 23], with *P* < 0.05 indicating statistical significance. All statistical tests were two-sided. The meta-regression analyses based on the mixed-effects model were used to assess whether the RRs of invasive fungal infections between prophylaxis with fluconazole and no prophylaxis could be predicted by the average dosage of fluconazole (mg/kg/day), which was multiplied by the dose of fluconazole (mg/kg) and the frequency of administration, and the total dosage of fluconazole (mg/kg) for the duration of prophylaxis, which was multiplied by the average dosage of fluconazole and the duration of prophylaxis (in days).

## Results

### Study selection

**Fig. 1:**
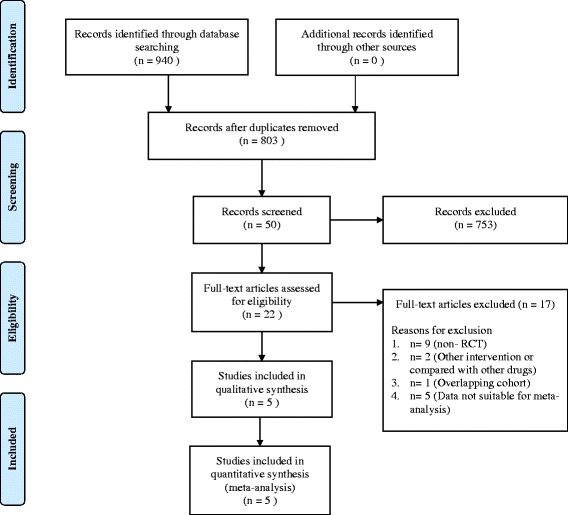
Flowchart of study selection

The literature search produced a total of 940 citations, including 50 that were considered potentially relevant (Fig. 1). Of these, 22 articles were considered to be of interest, and their full texts were retrieved for detailed evaluation. Seventeen of these 22 articles, including studies with other prophylactic agents, such as liposomal amphotericin B, were subsequently excluded, and the remaining five articles were included in the meta-analysis.

### Study characteristics

Five independent RCT studies enrolled 1006 preterm neonates, including 567 neonates assigned to the prophylaxis arm and 439 neonates assigned to the control arm [8–11, 17]. All qualified articles were published since 2001. Three RCTs were conducted in the USA, one was performed in Italy, and one was conducted in India. The quality of all of the trials was acceptable (Table 1). No evidence of publication bias was detected for the primary endpoint of this study (RR of invasive fungal infection) by either Begg’s or Egger’s test (Begg’s test, *p* = 0.4833; Egger’s test, *p* = 0.2795).

The five trials had different treatment patterns with different doses, frequencies and durations of fluconazole (Table 1). The study reported by Manzoni compared the outcomes of two prophylaxis regimens (3 or 6 mg/kg) with durations of 28 or 42 days according to the weight of the neonate in three arms [10]. All other studies had two arms with a prophylactic regimen (3 or 6 mg/kg). The duration of prophylaxis with fluconazole was 28 days in two studies and 42 days in two studies. The average dosage of fluconazole varied from 1.83 to 5 mg /kg/day, and the total dose of fluconazole for the duration of prophylactic treatment varied from 70 to 140 mg/kg. Efficacy data regarding invasive fungal infections and mortality were available for all of the included studies.

### Effects of the interventions

**Fig. 2:**
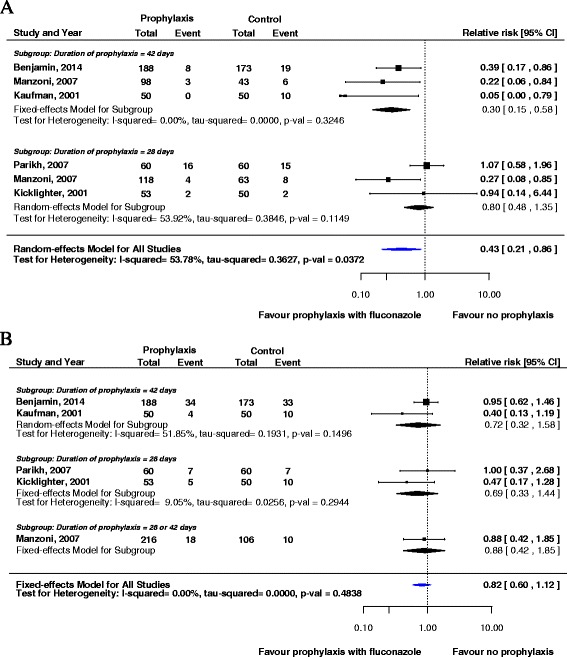
Forest plot of relative risks (RRs) between prophylaxis with fluconazole and no prophylaxis for invasive fungal infections (**a**) and mortality (**b**) categorized by the duration of prophylaxis (28 and 42 days). CI, confidence interval

Overall, compared with no prophylaxis, prophylaxis with fluconazole resulted in a significant reduction in invasive fungal infections (RR: 0.43 [95 % CI 0.21–0.86, *p* = 0.0179]) in the random-effects model (Fig. 2a). Categorized by the duration of prophylaxis, Fig. 2a also shows that compared with no prophylaxis, the combined RR for the subgroup administered prophylaxis for 42 days was 0.30 (95 % CI 0.15–0.58, *p* = 0.0004) in the fixed-effects model and that the combined RR for the subgroup administered prophylaxis for 28 days was 0.80 (95 % CI 0.48–1.35, *p* = 0.4048) in the random-effects model. The meta-analysis of the five studies showed that the RR of death prior to hospital discharge was 0.82 (95 % CI 0.60–1.12, *p* = 0.2093) in the group administered prophylaxis with fluconazole compared with the group not administered prophylaxis (Fig. 2b).

In the meta-regression analyzing the average dosage of fluconazole and the total dose of fluconazole for the duration of prophylaxis as independent variables and the log(RR) of invasive fungal infections as the response variable, neither the average dosage (*p* = 0.3973) nor the total dose for the duration of prophylaxis (*p* = 0.7089) was significantly associated with the log(RR) of invasive fungal infections.

## Discussion

This study extends the prior analyses regarding the choice between prophylaxis with fluconazole and no prophylaxis for the prevention of invasive fungal infections. The current meta-analysis demonstrates that the use of fluconazole for prophylaxis, compared with no prophylaxis, decreases the risk of invasive fungal infections in preterm infants in NICUs. These findings are consistent with the results of Austin’s study, which pooled data from seven trials and showed that the RR of invasive fungal infections between a systemic antifungal agent and no prophylaxis was 0.41 (95 %CI: 0.27 to 0.61) [15]. This effect was driven by the studies that employed a 42-day course of prophylaxis (RR: 0.30 ([95 % CI: 0.27–0.61]). A reduction of invasive candidiasis was not found in the subgroup of studies that used a shorter 28-day course of prophylaxis (RR: 0.80 [95 % CI: 0.48–1.35]). Because the mean length of stay in NICUs is greater than 40 days and preterm neonates in NICUs are at high risk for systemic fungal infections, the use of prophylactic fluconazole may be most effective within the NICU phase because it would prevent Candida colonization during the first 4 to 6 weeks of the neonates’ life [13, 24, 25]. This hypothesis may need to be tested through an RCT.

Regarding the dosing of prophylactic fluconazole, clinicians have raised concerns about the safety and cost of fluconazole prophylaxis treatment [12]. One pharmacokinetic analysis showed that a dose of 3 or 6 mg/kg twice weekly for early prevention during the first 42 days of life is equivalent to an area under the concentration curve (AUC) of 50 or 100 mg × h/L, respectively, and maintains fluconazole concentrations of at least 2 or 4 g/mL, respectively, for half of the dosing interval. For late prevention, a dose of 6 mg/kg every 72 h provides similar exposure as a daily dose of 3 mg/kg. These findings indicate that twice-weekly prophylaxis regimens can provide adequate serum levels for the prevention of invasive candidiasis when the unit-specific minimum inhibitory concentration (MIC) is taken into account [26, 27]. The current meta-regression suggests that the average dosage of fluconazole (mg/kg/day) and total dose of fluconazole (mg/kg) for the duration of prophylaxis are not significantly associated with the log(RR) of invasive fungal infections. These results are consistent with Manzoni’s findings, which showed no significant differences between the 6-mg and 3-mg arms [10]. Basically, the findings for these two factors similarly imply that the duration of prophylaxis appears to significantly influence the fluconazole effect. Furthermore, the low dosing of fluconazole indicates that the MICs of fungal strains causing colonization or infection significantly increased over a 10-year period in various NICUs [13].

Prophylactic fluconazole did not significantly reduce death before discharge. This finding is similar to those obtained in other meta-analyses [15, 28], but should be carefully explained because the mortality rates in the RCT studies that were included in the meta-analysis were ≤20 % and invasive fungal infections contributed to approximately 20 % of the mortalities [29]. The previous RCT studies may not have had sufficient power to detect the impact of prophylactic fluconazole on mortality associated with fungal infection due to their relatively small sample sizes (<200 infants in each arm) [8–11, 17]. The low baseline incidence of invasive candidiasis may contribute to the lack of a significant reduction in mortality mediated by a fluconazole-induced reduction in invasive infection. The non-RCT studies found that prophylactic fluconazole eliminated all Candida-related mortality in preterm infants in NICUs [24, 30].

The limitations of the current analysis should be noted. The first possible limitation is that this study did not compare the efficacy and safety of fluconazole with those of other systemic antifungal agents, such as nystatin, due to the wide usage of prophylactic fluconazole in NICUs. Specifically, more than 20 studies examined fluconazole prophylaxis in more than 5000 neonates [29]. Furthermore, this study did not include trials that investigated the efficacy and safety of prophylactic oral/topical non-absorbed antifungal agents to prevent invasive fungal infection in premature infants because of the methodological weaknesses of these trials [28]. The second limitation is the lack of individual-level data, which prohibited the evaluation of the associations between individual variables and the study outcomes. Instead, we used between-study meta-regressions when possible. Third, the results are limited to Western preterm neonates due to the absence of data on Eastern preterm neonates, and a recent systematic review found a higher incidence of *C. parapsilosis* in Australia and North America compared with Europe, as demonstrated by the selected studies from North America (11.4 %) and Europe (13.2 %) in the current meta-analysis [31]. The different epidemiology may have an impact on the clinical decision of prophylaxis [32]. Fourth, the safety profile of prophylactic fluconazole was not used as an outcome in the current meta-analysis because serious adverse events or fluconazole-related toxic effects are similar to those observed without prophylaxis. Fifth, the small sample size of the trials that were included in the current analysis may have impacted the trials’ ability to detect events related to fungal infections and mortality. This bias should be taken into account. Finally, this meta-analysis did not include neurodevelopmental impairment as an endpoint because multiple factors can affect neurodevelopmental impairment [33]. Further larger trials with long-term outcome data are needed.

## Conclusion

A six-week course of prophylactic fluconazole decreases late-onset invasive candidiasis. The mortality rate was slightly improved with prophylactic fluconazole compared with no prophylaxis, but the difference was not significant. By weighing the costs and health benefits, a low-intensity dosing and regimen of fluconazole may be employed. However, the clinical decision of administering prophylactic fluconazole routinely to preterm infants should be made based on the local setting, including the epidemiology. Further studies are needed to evaluate the effectiveness and safety of different regimens of fluconazole. Furthermore, the appropriate duration of treatment is an important issue that merits further evaluation through high-quality RCTs.

## Abbreviations

ELBW, extremely-low-birth-weight; NICUs, neonatal intensive care units; PRISMA, preferred reporting items for systematic reviews and meta-analyses; RCT, randomized controlled trial; RRs, relative risks; VLBW, very-low-birth-weight
